# HO-1-Enriched Lung-Derived Exosomes Mediate Cognitive Impairment in a Mice Model of COPD Exacerbation

**DOI:** 10.1155/carj/9976065

**Published:** 2025-11-28

**Authors:** Guang Yu Yin, Jia Qiang Zhang, Zhou Ling Xie, Yun Xia Jin, Yu Wang, Feng Heng Qian

**Affiliations:** The Second Department of Pulmonary and Critical Care Medicine, The First Affiliated Hospital of Kunming Medical University, Kunming, Yunnan, China

**Keywords:** cognitive dysfunction, COPD exacerbation mice, exosome, HO-1, lung–brain axis

## Abstract

**Objective:**

This study aimed to elucidate the impact of HO-1 on cognitive function in chronic obstructive pulmonary disease (COPD) exacerbation mice and uncover the bridging role of exosomes in the interorgan transport of HO-1 along the “lung–brain” axis, providing novel insights into COPD-associated cognitive dysfunction.

**Methods:**

HO-1(−/−) and wild-type mice were subjected to COPD exacerbation modeling through cigarette smoke and LPS exposure. Four groups were divided: (A) HO-1(+/+) mice; (B) HO-1(+/+) COPD exacerbation mice; (C) HO-1(−/−) mice; and (D) HO-1(−/−) COPD exacerbation mice. Cognitive function was assessed using the Morris water maze. Lung-derived exosomal HO-1 was quantified by Western blot. Fluorescently labeled exosomes from Groups A/B were injected into HO-1(−/−) mice via tail vein, generating four new cohorts: Group I: HO-1(+/+) mice injected with PBS; Group II: HO-1(−/−) mice injected with PBS; Group III: HO-1(−/−) mice injected with exosome (Exos) from Group A; Group IV: HO-1(−/−) mice injected with Exos from Group B; Exos biodistribution was tracked via in vivo imaging, followed by cognitive reassessment and HO-1 quantification.

**Results:**

Groups B/D showed reduced target quadrant dwell time versus Groups A/C, and Group B exhibited longer target quadrant dwell time than Group D. This shows that COPD exacerbation mice had cognitive decline, which was exacerbated by HO-1 deficiency. Group B exhibited higher HO-1 expression in lung exosomes than Group A. Injected exosomes accumulated preferentially in lungs over brain; Group IV displayed worse cognitive impairment than Group III.

**Conclusions:**

COPD exacerbation mice exhibit cognitive decline regardless of HO-1 expression status, but HO-1 knockout COPD exacerbation mice demonstrate more pronounced cognitive impairment. In COPD exacerbation mice, HO-1 expression is elevated in lung-derived exosomes. Cross-organ transduction experiments confirm that HO-1 can be transported via Exos and mediate cognitive dysfunction in COPD exacerbation mice. Thus, HO-1 exerts a concentration-dependent dual effect on brain function: protective at physiological levels yet detrimental when in excess.

## 1. Introduction

Chronic obstructive pulmonary disease (COPD) is associated with multiple extrapulmonary manifestations [1]. Cognitive impairment in COPD patients severely impacts quality of life and imposes substantial socioeconomic burdens [2]. Epidemiological studies reveal a 61% prevalence of cognitive decline (including memory, executive function, and attention deficits) in COPD populations [3], markedly higher than the 12% observed in age-matched healthy individuals [4]. While shared risk factors (e.g., smoking, hypoxemia, hypertension, and infections) partially explain COPD-cognition associations [5], independent links persist between pulmonary function decline [6], altered blood gas parameters [7], and cognitive deterioration.

Current mechanistic research focuses on energy metabolism dysregulation and hypoxia/hypercapnia-mediated neurotoxicity. Chronic hypoxia in COPD suppresses mitochondrial oxidative phosphorylation, forcing neurons to rely on inefficient glycolysis [8]. Hypoxia upregulates transferrin receptor 1 (TfR1), promoting aluminum accumulation in gray matter and accelerating tau phosphorylation/β-amyloid (Aβ) deposition [9]. Concurrent hypercapnia exacerbates cognitive outcomes through blood hyperviscosity, cerebral hypoperfusion, and excitotoxicity [7]. Heck et al. [10] proposed novel insights into respiratory rhythm-mediated gamma oscillation modulation of cognitive-affective circuits, expanding COPD-related cognitive research. However, cognitive decline persists in nonhypoxemic COPD patients, suggesting mechanisms beyond gas exchange abnormalities. Systemic inflammation—a hallmark of COPD [11]—enables circulating cytokines to compromise blood–brain barrier (BBB) integrity, driving neuroinflammation and neuronal dysfunction [12]. Proinflammatory cytokines activate JNK/NF-κB signaling to inhibit IRS-1 phosphorylation and GLUT4 activity, inducing neuronal insulin resistance [13]. Peripheral inflammation also shifts tryptophan metabolism toward kynurenine pathways, depleting neurotransmitter precursors [14]. Thus, deciphering multiorgan inflammatory crosstalk is critical for understanding COPD-associated cognitive impairment.

Heme oxygenase-1 (HO-1), a redox-regulatory enzyme induced by inflammatory stimuli [15], exhibits low basal expression but strong upregulation under oxidative/inflammatory stress [16]. HO-1 catalyzes heme degradation into iron, biliverdin, and carbon monoxide (CO)—the latter two exerting anti-inflammatory/vasodilatory and antioxidant effects, respectively. Some previous studies conducted by our team have found that HO-1 plays a crucial antioxidant role in COPD [17, 18]. Wang et al. [19] measured the HO-1 activity, HO-1 protein expression, and HO-1 mRNA expression in the lung tissues of the COPD group, the COPD complicated with lung cancer group, the lung cancer group, and healthy individuals. They found that the expression of HO-1 in the lung tissues of COPD patients was significantly increased.

HO-1 also plays a crucial role in tissue pathological changes such as brain injury [20]. A large number of studies have confirmed the protective effect of HO-1 in the nervous system under various oxidative stress conditions. The typical functions of HO-1 in the brain include antioxidant, anti-apoptotic, vasodilatory, and anti-inflammatory responses [21, 22]. Under physiological conditions, HO-1 is barely detectable in most cell types of the healthy brain. However, in neurons, astrocytes, and microglia of Alzheimer's disease [23], Parkinson's disease [24], and multiple sclerosis, which mostly occur in the elderly [25], the low expression of HO-1 increases significantly under pathological stimulation. Some researchers believe that this dose-dependent increase of HO-1 is to protect the brain from oxidative damage [26]. However, more than 53 studies have revealed the cytotoxic effect of HO-1 in Alzheimer's disease, such as abnormal iron deposition in astrocytes [27], nerve damage [28], and the uptake of excitotoxic neurotransmitters from the synaptic cleft [29], leading to cognitive decline, olfactory impairment, and neuronal endocrine disorders. Currently, more studies tend to suggest that HO-1 has dual effects of cytoprotection and cytotoxicity in the development of neurodegenerative diseases, and its functional activity needs to be precisely regulated. Many drugs regulate oxidative stress by inducing HO-1 expression, but the cytotoxic mechanism of HO-1 on glial cells remains to be further studied [30].

COPD, characterized by systemic inflammation, extends beyond pulmonary manifestations with underappreciated cerebral involvement. Emerging evidence suggests complex lung–brain interactions mediated through both direct inflammatory cascades and indirect pathways. The novel “lung–brain axis” concept redefines these organs as interconnected functional units maintaining dynamic bidirectional communication via sophisticated biomolecular networks, rather than isolated systems [31]. This paradigm shift underscores the necessity to investigate critical mediators like HO-1 in COPD-associated inflammation and their potential interorgan signaling mechanisms. Interorgan communication typically relies on molecular mediators such as nucleic acids and proteins. However, the BBB—a selective semipermeable interface between cerebral microvasculature and neural tissue—substantially restricts macromolecular transit [32]. As one of the most sophisticated physiological barriers [33], the BBB actively regulates molecular permeability, balancing neuroprotection against toxic substances with nutrient transport according to cerebral metabolic demands. Intriguingly, exosome (Exos) lipid bilayer extracellular vesicles (40–160 nm diameter) containing proteins, nucleic acids, and metabolites represent exceptional BBB-penetrating vectors [34]. These endogenous nanocarriers mediate intercellular communication through targeted biomolecule delivery [35], with emerging applications as CNS-targeted drug delivery platforms [36, 37]. Bioinformatic analysis of the EVpedia database reveals significant HO-1 enrichment within exosomal cargos [38], suggesting their potential role in transporting HO-1 to distal organs, including the brain. This Exos-mediated signaling mechanism may critically influence cognitive function through targeted molecular delivery. Elucidating the molecular foundations of lung–brain axis communication, particularly Exos-mediated signal transduction, could provide pivotal insights for intervening in COPD-related neurodegenerative processes.

Based on the aforementioned theories, we hypothesize that under direct smoke stimulation, the abnormally elevated HO-1 in lung tissues of COPD patients may not solely exert localized effects within the lungs. Substantial HO-1 overexpression might be transported via Exos through the lung–brain axis, subsequently modulating redox homeostasis and inflammatory microenvironments in the central nervous system, ultimately contributing to the pathological progression of COPD-associated cognitive impairment.

This study employs HO-1 knockout mice to experimentally compare the impact of HO-1 on cognitive function in COPD exacerbation mice and assess HO-1 expression profiles in lung-derived Exos. Furthermore, we establish an Exos-mediated HO-1 cross-organ transduction model at the animal level to investigate interorgan communication between the lung and brain.

## 2. Materials and Methods

### 2.1. Experimental Animals

Six HO-1(±) heterozygous mice (three males and three females) were purchased from Cyagen Model Organism Research Center (Taicang) Co., Ltd. (Production License: SCXK(Su) 2018-0003). Mice were housed under standard conditions (23°C–26°C, 30%–50% humidity) with free access to food/water. After 12 months of breeding, 28 HO-1(−/−) homozygous mice were genotyped by PCR electrophoresis. Additional C57BL/6J mice were obtained from the Kunming Medical University Animal Center (License: SCXK(X) 2020-0004). A total of 38 HO-1(+/+) and 21 HO-1(−/−) mice were ultimately included in the study. All procedures were approved by Kunming Medical University Animal Ethics Committee.

### 2.2. COPD Exacerbation Modeling and Grouping

COPD was induced via cigarette smoke (CS) exposure combined with LPS nasal instillation. Mice received 30 μg/6 μL LPS on days 1, 25, and 50. Daily CS exposure (10 cigarettes, 30 min/session, twice daily, 5 days/week) was administered in a custom glass chamber for 8 weeks. Four groups were established:

• **Group A**: HO-1(+/+) mice, wild-type control (WT-Con, *n* = 19)
• **Group B**: HO-1(+/+) COPD exacerbation mice, wild-type COPD model (WT-COPD, *n* = 19)
• **Group C**: HO-1(−/−) mice, HO-1 knockout control (KO-Con, *n* = 11)
• **Group D**: HO-1(−/−) COPD exacerbation mice, HO-1 knockout COPD model (KO-COPD, *n* = 11)

Three animals per group underwent comprehensive model validation through (1) gross morphological observation; (2) histopathological analysis of lung tissue sections; and (3) serum quantification of COPD biomarkers.

### 2.3. Morris Water Maze

Eight mice per group underwent cognitive testing. A circular pool (25°C water) was used for 5-day training (platform visible), followed by a probe test (platform removed) on Day 6. Target quadrant dwell time and platform crossings were recorded.

### 2.4. Lung Exos Isolation

Pulmonary Exos were isolated from mouse lung tissues using a tissue-specific Exos isolation kit (Umibio, Cat# UR52163) according to the manufacturer's instructions. In brief, approximately 1 g of lung tissue from each mouse was minced into 1 mm^3^ fragments, rinsed with PBS, and homogenized in Solution A2. The homogenate was incubated for 20 min at 37°C with continuous shaking at 80 rpm. After centrifugation at 10,000 × g for 10 min at 4°C, the supernatant was collected and mixed with one-fourth volume of Solution B2, followed by 10 μL of PKH26 dye for labeling. The mixture was incubated at 4°C for 30 min and centrifuged again at 10,000 × g for 20 min. The pellet was resuspended in PBS and further purified using a size-exclusion column (Umibio).

Due to the limited Exos yield from individual mouse lungs, a sample pooling strategy was employed: Tissues from two to three mice within the same experimental group were combined to form one biological replicate. Three such replicates were prepared per group (*n* = 8 mice/group in total) to ensure sufficient material and statistical reliability.

### 2.5. Exosomal HO-1 Quantification

**Figure 1:**
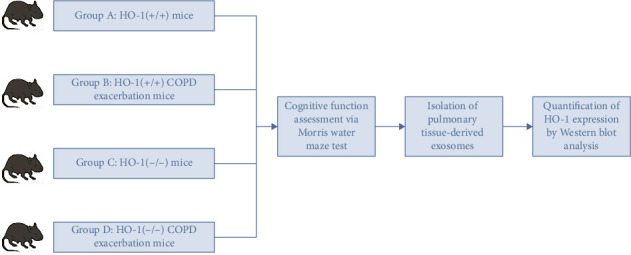
Experimental grouping and experimental flowchart.

Exos were lysed in RIPA buffer, and protein concentrations were measured spectrophotometrically. Equal loads (60 μg) were separated by SDS-PAGE, transferred to polyvinylidene fluoride (PVDF) membranes, and probed with HO-1 antibodies (β-actin as a loading control). Bands were visualized via ECL and analyzed using Image Master 3.0. The experimental workflow described above is schematically illustrated in Figure 1.

### 2.6. Exos Quantitative Detection

The concentration of the isolated Exos from the remaining eight mice in each of the WT-Control and WT-COPD groups was determined using the EXOCET Exosome Quantitation Assay Kit (System Biosciences, #EXOCET96A-1) according to the manufacturer's instructions. This assay quantifies Exos-specific esterase activity and provides a measurement that has been calibrated to particle count via NanoSight analysis by the manufacturer, as stated in the product manual. The final extracts were diluted to a uniform concentration in PBS for subsequent in vivo injections to ensure that each mouse received an equal particle number of Exos.

### 2.7. HO-1 Detection in Blood/Brain/Lung Tissues

**Figure 2:**
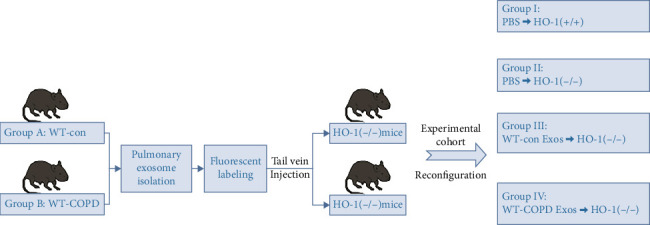
Schematic diagram of the establishment of the cross-organ transduction model.

The remaining eight mice in Groups A and B underwent pulmonary Exos isolation and quantitation using the aforementioned tissue-specific extraction kit. PKH26-labeled Exos (100 μg in 200 μL PBS per mouse) [39] were administered via tail vein injection to HO-1(−/−) mice. Control configurations included:

• Group I: HO-1(+/+) mice injected with PBS;
• Group II: HO-1(−/−) mice injected with PBS;
• Group III: HO-1(−/−) mice injected with Exos from WT-Con;
• Group IV: HO-1(−/−) mice injected with Exos from WT-COPD;

All groups maintained *n* = 8 biological replicates. Exos biodistribution was tracked using a small-animal in vivo imaging system at Day 1 and Day 5 postinjection. Cognitive reassessment was subsequently performed through Morris water maze testing. The experimental design is schematized in Figure 2.

### 2.8. Detection of HO-1 Carried by Exos in Blood, Brain, and Lung Tissues of Mice Postinjection

**Figure 3:**
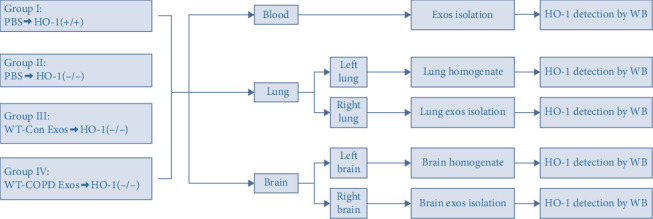
Schematic workflow for HO-1 detection in blood, lung, and brain tissues.

Mice from Groups I–IV were anesthetized with 1% pentobarbital (0.1 mL/10 g), followed by retro-orbital blood collection for plasma Exos. After cardiac perfusion with saline, lungs and brains were harvested and bisected: left lobes/hemispheres for tissue homogenates (8 replicates/group), right portions for Exos isolation. Due to limited Exos yields from blood/right lung/right brain, pooled samples (2–3 mice/group) were analyzed in triplicate for HO-1 by Western blot, as shown in Figure 3.

### 2.9. Statistical Analysis

Normality (Shapiro–Wilk) and variance homogeneity (Levene's test) were verified. Parametric data (*t*-test/ANOVA) and nonparametric data (Mann–Whitney *U*) were analyzed in SPSS 19.0. Graphs were generated using Prism 10.0.

## 3. Results

### 3.1. General Observations and Lung Histopathology

**Figure 4:**
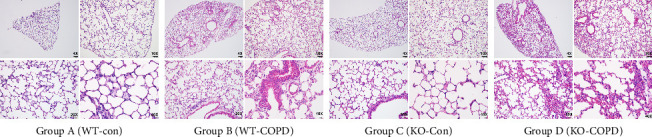
HE staining of lung tissue sections of mice in each group.

Non-COPD groups (A/C) exhibited normal fur gloss, regular respiration, and active behavior without mortality. COPD exacerbation groups (B/D) showed dull fur, partial alopecia, agitation, huddling during smoke exposure, lethargy, abdominal distension, tachypnea, and head-nodding movements. Histopathological analysis (Figure 4) revealed intact alveolar structures and ciliary arrangements in Groups A/C. COPD exacerbation groups demonstrated collapsed alveolar walls, emphysematous bullae, inflammatory infiltrates, epithelial shedding, and narrowed airways, consistent with COPD pathology.

### 3.2. Serum TNF-α and IL-18 Levels

**Figure 5:**
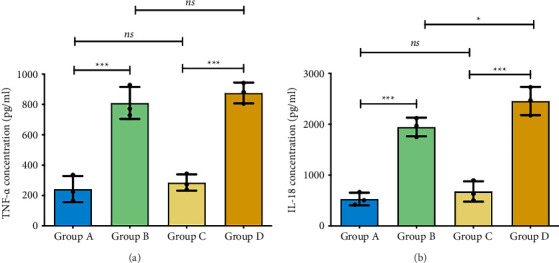
Serum levels of TNF-α and IL-18 in mice across different experimental groups. (A) TNF-α and (B) IL-18 concentrations in serum were measured by ELISA. Group A: WT-Con, Group B: WT-COPD, Group C: KO-Con, Group D: KO-COPD. ^∗^, ^∗∗∗^represent statistical significance at the *p < *0.05, *p < *0.001 levels, respectively. “ns” indicates *p* > 0.05, indicating no significant difference between groups. (A) TNF-α concentration is elevated in COPD exacerbation mice. (B) IL-18 was elevated in COPD exacerbation mice and was higher in KO-COPD than in WT-COPD groups.

COPD exacerbation groups (B/D) displayed significantly elevated TNF-α and IL-18 versus controls (*p* < 0.001). Group D showed higher IL-18 than Group B (*p* < 0.05), though TNF-α levels were comparable. No differences existed between Groups A/C (Figure 5).

### 3.3. Morris Water Maze Performance

**Figure 6:**
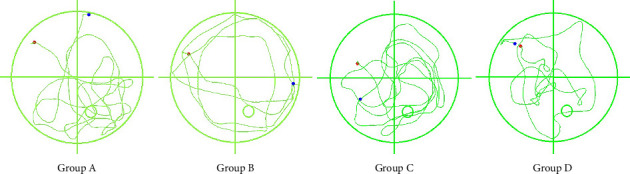
Mouse swimming paths during the Morris water maze probe trial. Group A: WT-Con, Group B: WT-COPD, Group C: KO-Con, Group D: KO-COPD. The solid circle shows the former platform location. Note the less focused search pattern in Group D/C.

**Figure 7:**
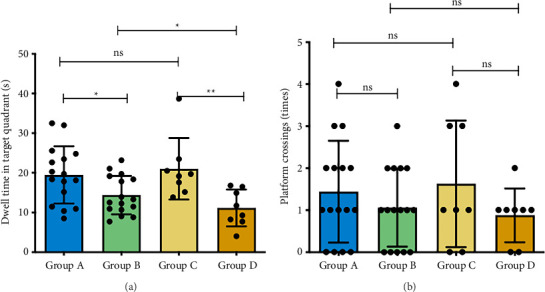
Spatial memory performance in the Morris water maze probe trial. Group A: WT-Con, Group B: WT-COPD, Group C: KO-Con, Group D: KO-COPD. ^∗^, ^∗∗^represent statistical significance at the *p < *0.05, *p < *0.01 levels, respectively. “ns” indicates *p* > 0.05, indicating no significant difference between groups. (a) The dwell time in the target quadrant was lower in the COPD exacerbation model groups than in the non-COPD groups, and it was further reduced in the KO-COPD group compared to the WT-COPD group. (b) No significant difference was found in the number of platform crossings among all groups.

Groups A/C exhibited longer target quadrant dwell times than B/D (*p*_AB_ < 0.05; *p*_CD_* *<* *0.01). Platform crossings showed no COPD-related differences (*p* > 0.05). Group B demonstrated longer dwell times than Group D (*p < *0.05), while HO-1 status in non-COPD mice (A/C) had no cognitive impact (Figures 6 and 7).

### 3.4. HO-1 Expression in Lung Exos

**Figure 8:**
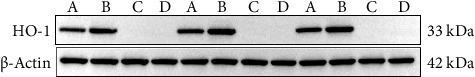
Detection of HO-1 in lung tissue-derived exosomes by Western blot analysis. Shown are representative protein bands from each experimental group.

**Figure 9:**
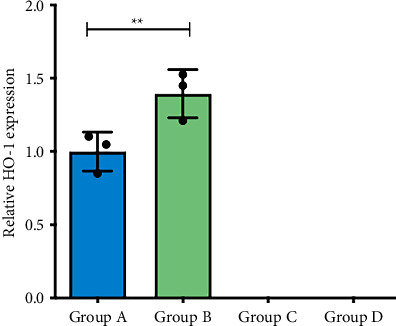
Detection and comparison of HO-1 expression levels in lung tissue-derived Exos across experimental groups. The WT-COPD group exhibited significantly higher exosomal HO-1 levels than the WT-Control mice. ^∗∗^*p < *0.01.

HO-1 knockout mice (Groups C and D) exhibited complete absence of HO-1 immunoreactivity across triplicate experiments (Figure 8). In Groups A and B, the normalized HO-1 values (*n* = 3 replicates) violated assumptions of normality and homogeneity of variance (detailed metrics in Table 1). Consequently, nonparametric Mann–Whitney U analysis revealed significantly elevated exosomal HO-1 cargo in Group B pulmonary Exos compared to Group A (*p < *0.01), as shown in Figure 9.

### 3.5. Exos Quantitative Detection

**Figure 10:**
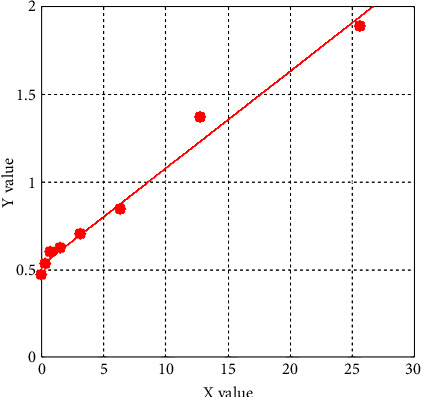
Standard curve for exosome quantification. A standard curve was generated by assaying serial dilutions of the provided exosome standard. The absorbance (OD) values exhibited a strong linear relationship with exosome concentration, as defined by the equation y = 0.06239x + 0.52856 (R^2^ = 0.98452), where “y” is the OD_405_ value and “x” is the exosome concentration (μg/μL). The concentrations of unknown samples were calculated by interpolating their OD values into this equation. The high *R*^2^ value indicates an excellent linear fit, confirming the reliability of the quantitation method used to determine exosome concentrations in experimental samples.

We quantified the yield of EVs isolated from the lung tissues of WT-Control and WT-COPD exacerbation mice using an Exos-specific esterase activity assay.  Table 2 shows the concentration of Exos isolated from mouse lung tissues. When these concentrations were plotted against the standard curve in Figure 10, we found that the total Exos yield was higher in the WT-Con group than in the WT-COPD group. Consequently, all Exos samples were adjusted to the same concentration prior to intravenous injection to ensure that any observed biological effects were due to the qualitative differences in the Exos (e.g., cargo like HO-1) rather than quantitative differences in particle number.

### 3.6. In Vivo Exos Tracking

**Figure 11:**
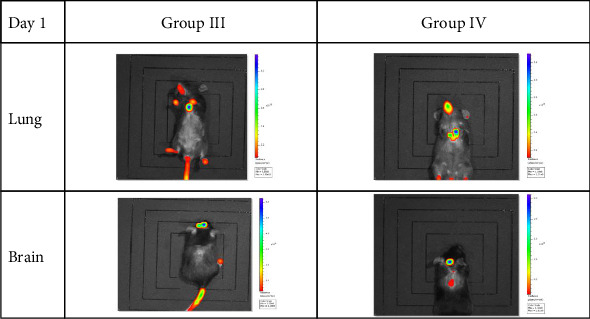
In vivo fluorescence imaging on Day 1 postinjection reveals distinct biodistribution of exosomes. Group I: HO-1(+/+) + PBS; Group II: HO-1(−/−) + PBS; Group III: HO-1(−/−) + WT-Con Exos; Group IV: HO-1(−/−) + WT-COPD Exos. HO-1 knockout mice were intravenously injected with PKH26-labeled Exos derived from the lungs of WT-Con or WT-COPD exacerbation mice. Representative images show that exosomes from WT-COPD exacerbation mice (Group IV) exhibited enhanced accumulation in the lungs and brain compared to those from WT-Control mice (Group III).

**Figure 12:**
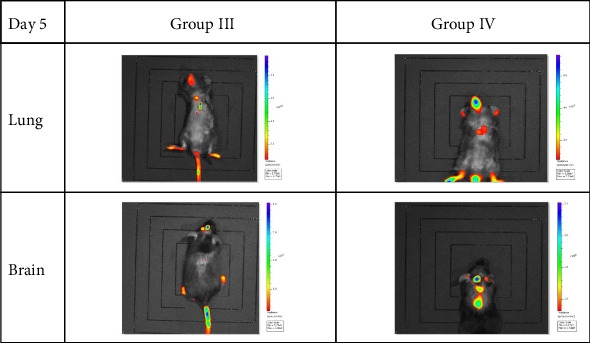
Sustained biodistribution of exosomes on Day 5 postinjection. In vivo fluorescence imaging reveals that the differential biodistribution patterns observed on Day 1 were maintained. Exosomes derived from WT-COPD exacerbation mice (Group IV) continued to show enhanced and sustained retention in the lungs and brain compared to those from WT-Control mice (Group III), which exhibited decreased signal intensity over time.

**Figure 13:**
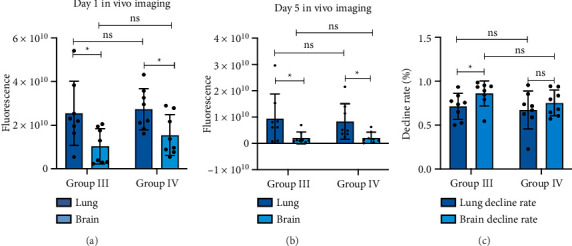
Biodistribution and clearance kinetics of lung-derived exosomes. Group I: HO-1(+/+) + PBS; Group II: HO-1(−/−) + PBS; Group III: HO-1(−/−) + WT-Con Exos; Group IV: HO-1(−/−) + WT-COPD Exos. (A, B) Fluorescence intensity showing higher and more sustained signals in lung tissue compared to brain tissue for both exosome types. (C) Quantification of the signal decline rate from Day 1 to Day 5, revealing significantly faster clearance from the brain in Group III. ^∗^*p* < *0.05.*

Mice injected with fluorescently labeled lung-derived Exos (Groups III and IV) showed fluorescence imaging in both the lungs and brains on Day 1 and Day 5 (as shown in Figures 11 and 12, with representative imaging screenshots of some mice from each group selected). On both Day 1 and Day 5, lung tissue fluorescence values were significantly higher than those in brain tissue (*p < *0.05). After 5 days, fluorescence values in both lungs and brains decreased. There was no statistically significant difference in fluorescence values between the lungs and brains of mice in Groups III and IV (*p* > 0.05). Using formula (1), the fluorescence value decline rates in the lungs and brains of the two groups were calculated on Day 1 and Day 5. Statistical analysis of these decline rates revealed that in Group III, the decline rate in the brain was significantly higher than that in the lung (*p < *0.05), while no significant difference was observed between the lung and brain decline rates in Group IV. Additionally, there were no significant differences in the decline rates of lung or brain tissues between Group III and Group IV (*p* > 0.05). The statistical results are presented in Figure 13.

Decline rate (%) = (Fluorescence value on Day 1—Fluorescence value on Day 5)/Fluorescence value on Day 1 × 100% (1)

### 3.7. Postinjection Cognitive Assessment

**Figure 14:**
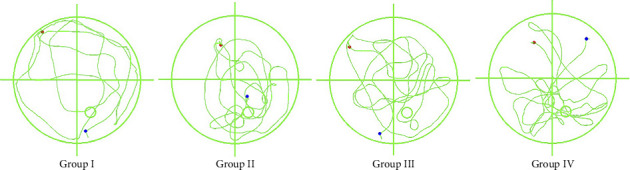
Mouse swimming paths during the Morris water maze probe trial following exogenous injections. Group I: HO-1(+/+) + PBS, Group II: HO-1(−/−) + PBS, Group III: HO-1(−/−) + WT-Con Exos, Group IV: HO-1(−/−) + WT-COPD Exos. The solid circle indicates the former platform location.

**Figure 15:**
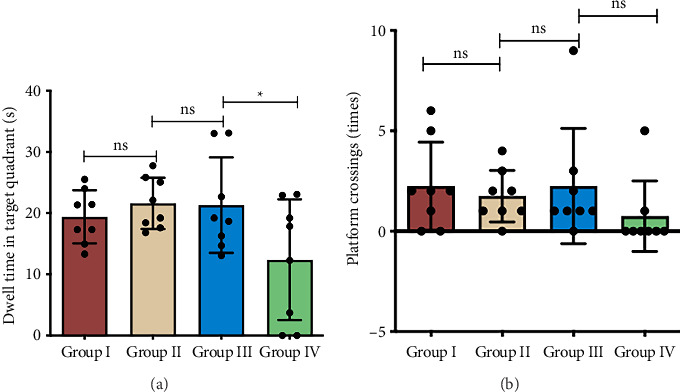
Spatial memory assessment after exogenous exosome injection. Group I: HO-1(+/+) + PBS, Group II: HO-1(−/−) + PBS, Group III: HO-1(−/−) + WT-Con Exos, Group IV: HO-1(−/−) + WT-COPD Exos. ^∗^represents statistical significance at the *p < *0.05. “ns” indicates *p* > 0.05. (a) Dwell time in the target quadrant was significantly lower in Group IV than in Group III. (b) No significant difference was observed in the number of platform crossings among all groups.

Figure 14 displays representative Morris water maze tracking plots from subset cohorts, with statistical profiles detailed in Figure 15. Group IV exhibited significantly reduced dwell time in the target quadrant compared to Group III (*p < *0.05), while platform crossing frequency showed no inter-group difference (*p* > 0.05). No significant inter-group differences in either parameter were observed among Groups I, II, and III (*p* > 0.05).

### 3.8. HO-1 Distribution Across Tissues

**Figure 16:**
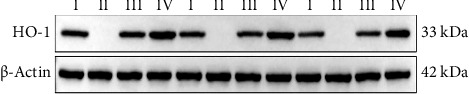
Detection of HO-1 in blood-derived exosomes by Western blot analysis. Group I: HO-1(+/+) + PBS, Group II: HO-1(−/−) + PBS, Group III: HO-1(−/−) + WT-Con Exos, Group IV: HO-1(−/−) + WT-COPD Exos. Shown are representative protein bands from each experimental group; this is a representative blot showing qualitative expression patterns. For quantitative analysis, see Figure 20.

**Figure 17:**
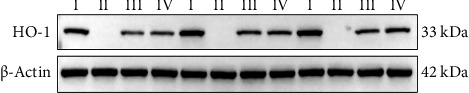
Detection of HO-1 in left lung tissue homogenates by Western blot analysis. Groups: I: HO-1(+/+) + PBS; II: HO-1(−/−) + PBS; III: HO-1(−/−) + WT-Con Exos; IV: HO-1(−/−) + WT-COPD Exos. Shown are representative protein bands from each experimental group.

**Figure 18:**
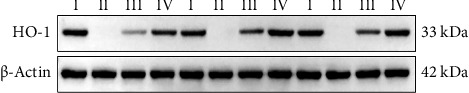
Detection of HO-1 in right lung-derived exosomes by Western blot analysis. Groups: I: HO-1(+/+) + PBS; II: HO-1(−/−) + PBS; III: HO-1(−/−) + WT-Con Exos; IV: HO-1(−/−) + WT-COPD Exos. Shown are representative protein bands from each experimental group.

**Figure 19:**
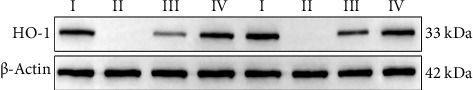
Detection of HO-1 in left brain tissue homogenates by Western blot analysis. Groups: I: HO-1(+/+) + PBS; II: HO-1(−/−) + PBS; III: HO-1(−/−) + WT-Con Exos; IV: HO-1(−/−) + WT-COPD Exos. Shown are representative protein bands from each experimental group.

**Figure 20:**
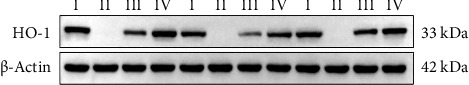
Detection of HO-1 in right brain-derived exosomes by Western blot analysis. Groups: I: HO-1(+/+) + PBS; II: HO-1(−/−) + PBS; III: HO-1(−/−) + WT-Con Exos; IV: HO-1(−/−) + WT-COPD Exos. Shown are representative protein bands from each experimental group.

**Figure 21:**
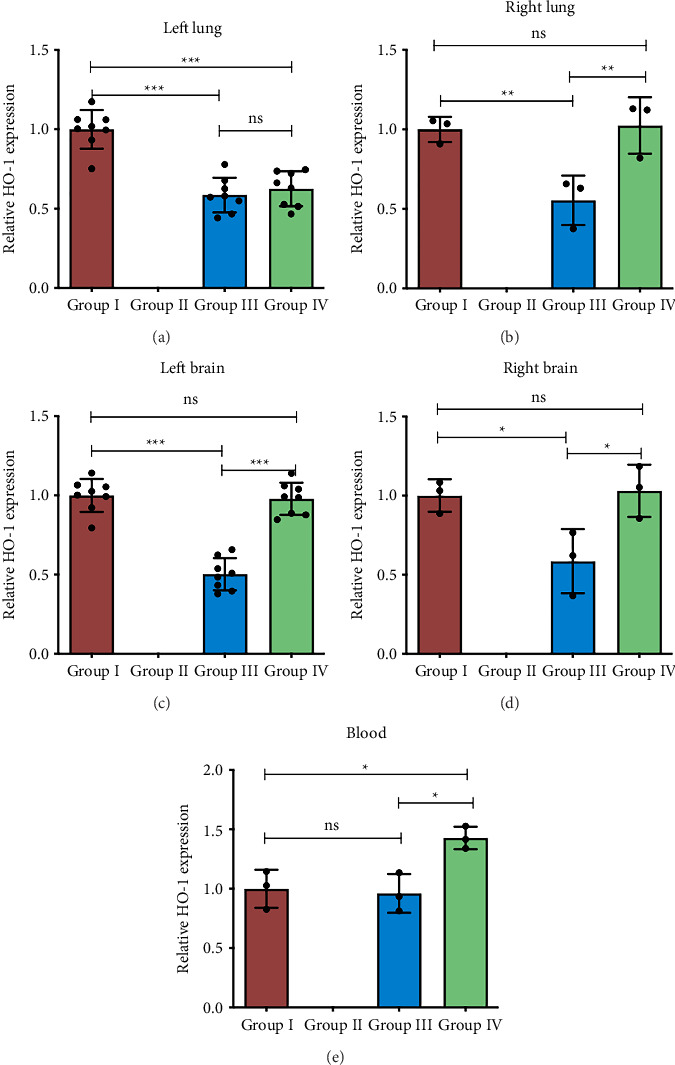
HO-1 expression is differentially regulated across tissues following exogenous exosome injection. Groups: I: HO-1(+/+) + PBS; II: HO-1(−/−) + PBS; III: HO-1(−/−) + WT-Con Exos; IV: HO-1(−/−) + WT-COPD Exos. ^∗^, ^∗∗^, ^∗∗∗^represent statistical significance at the *p < *0.05, *p < *0.01, and *p < *0.001 levels, respectively. “ns” indicates *p* > 0.05. (a) HO-1 protein levels in left lung homogenates. Group I showed higher expression than Groups III and IV. (b) HO-1 levels in right lung-derived exosomes. Expression was higher in Groups I and IV compared to Group III. (c) HO-1 levels in left brain homogenates. Expression was higher in Groups I and IV compared to Group III. (d) HO-1 levels in right brain-derived exosomes. Expression was higher in Groups I and IV compared to Group III. (e) HO-1 levels in blood-derived exosomes. Group IV showed higher expression than Groups I and III.

Figures 16, 17, 18, 19 and 20 present Western blot analyses of HO-1 expression in blood, lung, and brain tissues across Groups I–IV. Due to the use of distinct PVDF membranes for different tissues, inter-tissue densitometric comparisons were technically infeasible. Semi-quantitative analysis of normalized HO-1 levels (grayscale ratios) revealed comparable baseline HO-1 distribution in blood, pulmonary tissues, and brain-derived Exos of wild-type C57 mice (without statistical verification). When HO-1 content increased in vivo, HO-1 expression in lung tissue, lung-derived Exos, brain tissue, brain-derived Exos, and blood Exos all elevated. Statistical comparisons between different tissues in each group are shown in Figure 21. In Group IV mice, HO-1 expression in blood, lung-derived Exos, brain tissue, and brain-derived Exos was significantly higher than that in Group III mice (*p*_blood_ < 0.05, *p*_*right*⁣*lung*⁣*Exos*_ < 0.01, *p*_*left*⁣*brain*⁣*homogenate*_ < 0.001, *p*_right⁣brain⁣Exos_ < 0.05). Except for lower HO-1 expression in left lung homogenate compared to Group I (*p < *0.001) and higher HO-1 in blood Exos than Group I (*p* < 0.05), relative HO-1 expression in other tissues of Group IV mice showed no significant differences from Group I. This suggests that HO-1 content in blood, lung-derived Exos, and brain tissue of HO-1-positive COPD exacerbation mice might be twice or more that of normal non-COPD exacerbation mice. In Group III, HO-1 expression in blood Exos showed no significant difference from Group I (*p* > 0.05), while HO-1 expression in left/right lung tissues and their Exos, as well as left/right brain tissues and their Exos, was lower than Group I (*p*_*left*⁣*lung*⁣*homogenate*_ < 0.001, *p*_right⁣lung⁣Exos_ < 0.01, *p*_left⁣brain⁣homogenate_ < 0.001, *p*_right⁣brain⁣Exos_ < 0.05). From the statistical histograms, no significant difference was observed in relative HO-1 expression between left lung homogenate and right lung-derived Exos in Group III mice. In Group IV mice, HO-1 expression detected in right lung-derived Exos was higher than that in left lung homogenate, while relative HO-1 expression in left and right brain tissues showed comparable levels. However, due to samples from blood, left lung, left brain, right lung, and right brain not being on the same PVDF membrane, this conclusion could not be further statistically analyzed.

## 4. Discussion

COPD is strongly associated with increased risk of mild cognitive impairment (MCI) [40]. Two epidemiological studies demonstrated an approximately 80% elevated 5- and 25-year MCI risk in COPD patients [41, 42], which aligns with our murine model findings: COPD exacerbation mice (Groups B/D) exhibited reduced target quadrant dwell times in Morris water maze tests compared to non-COPD controls (Groups A/C), confirming COPD as an independent risk factor for cognitive decline. Notably, the pathological interplay between COPD and cognitive dysfunction involves multidimensional mechanisms, including systemic inflammation, mitochondrial dysfunction, and redox imbalance. While these interactions may obscure HO-1's biological effects, our cross-organ transduction experiments unequivocally established HO-1's critical role in cognitive regulation.

HO-1 is widely recognized as a cytoprotective factor primarily exerting antioxidant effects [43]. Its expression is tightly regulated at the transcriptional level [44], with multiple transcription factors binding to the 5′-untranslated region (5′-UTR) of the HO-1 gene [45], including Nrf2 [46], HIF-1 [47], and NF-κB [48]. Additionally, HO-1 expression is modulated by signaling cascades such as MAPK and PI3K/Akt [49]. The systemic significance of HO-1 in homeostasis and iron balance was inferred from early studies on HO-1-deficient mice, which exhibited systemic iron dysregulation characterized by hepatic/renal iron deposition and anemia [50]. HO-1-deficient mice and their endothelial cells demonstrate heightened sensitivity to oxidative stress [51]. Lee and Chau further confirmed that inhibiting HO-1 enzymatic activity abolishes the anti-inflammatory effects of IL-10 in LPS-stimulated macrophages, leading to increased TNF-α production [52]. Cumulative evidence highlights the pleiotropic anti-inflammatory roles of HO-1 through diverse molecular mechanisms [53]. Similarly, current research emphasizes HO-1's antioxidative protective role in COPD lung tissues. For instance, agents such as carotenoids [54], aucubin [55], and alantolactone [56] significantly upregulate pulmonary HO-1 expression via Nrf2 activation, suppressing alveolar macrophage inflammation and delaying emphysema progression. These findings align with our observation of milder pulmonary inflammation in HO-1(+/+) COPD exacerbation mice compared to HO-1(−/−) counterparts. Notably, in the Morris water maze test, HO-1(+/+) COPD exacerbation mice exhibited better cognitive preservation than HO-1(−/−) COPD exacerbation mice, suggesting HO-1-mediated neuroprotection in the brain. However, this protective effect is context-dependent: No cognitive differences were observed between HO-1(−/−) and HO-1(+/+) groups in non-COPD models. We hypothesize a “threshold effect”—HO-1 exerts cytoprotective antioxidant effects only within specific concentration ranges, beyond which neurotoxicity predominates. Under non-COPD physiological conditions, baseline oxidative stress levels are low, and HO-1's antioxidant effects may be fully compensated by endogenous defense systems (e.g., superoxide dismutase [SOD], glutathione) [57], resulting in no significant cognitive impact of HO-1 presence or absence.

Growing evidence reveals the dual roles of HO-1 in neurodegenerative diseases. On one hand, beyond its capacity to convert pro-oxidant heme into antioxidant degradation products, HO-1 induction in Parkinson's disease enhances α-synuclein proteasomal degradation, prevents dopaminergic neuron death by promoting neurotrophic factor synthesis, and augments antioxidant responses [58–60]. On the other hand, HO-1 overexpression in astrocytes exacerbates neuronal injury by oxidizing cholesterol to oxysterols and elevating intracellular cholesterol levels while reducing oxysterol concentrations in rats [61]. Studies suggest that HO-1's cytoprotective or cytotoxic effects in neuropathogenesis may depend on distinct signaling pathways: Nrf2-dependent HO-1 activation confers neuroprotection, whereas AP-1- or NF-κB-driven HO-1 induction exerts cytotoxicity in the CNS [30]. Additionally, dysregulation of HO-1-catalyzed heme degradation may disrupt iron metabolism, leading to neurodegeneration in neurons and glia. Iron dyshomeostasis likely bridges HO-1's dual roles and cognitive impairment.

Fe^2+^, a critical byproduct of HO-1 activity, physiologically regulates cellular respiration, oxygen metabolism, signal transduction, energy metabolism, and DNA synthesis/repair. However, pathological Fe^2+^ overload triggers ROS bursts via Fenton reactions, inducing lipid peroxidation and ferroptosis [62, 63]—a regulated cell death modality defined by the Nomenclature Committee on Cell Death as oxidative microenvironmental alterations constitutively controlled by glutathione peroxidase 4 (GPX4), which is inhibitable by iron chelators and lipophilic antioxidants [64]. Recent advances implicate ferroptosis as a key driver of neuronal death in Parkinson's, Alzheimer's, and Huntington's diseases [65, 66]. Ferroptosis is regulated by GPX4, lipid synthesis, iron metabolism, and the Nrf2 pathway [63, 67, 68], with elevated intracellular iron levels heightening susceptibility [69]. Consequently, HO-1 overexpression expands labile iron pools, precipitating ferroptosis [70] and subsequent tissue dysfunction.

The lungs, as primary targets of environmental toxins, exhibit marked HO-1 upregulation in specific cell types (e.g., alveolar macrophages, type II pneumocytes) under oxidative stress, a phenomenon validated across multiple COPD animal models [71]. Notably, Maestrelli et al.'s clinical study revealed disease-specific HO-1 expression: COPD patients showed significantly increased HO-1-positive alveolar macrophages compared to healthy nonsmokers, whereas smokers (with or without COPD) exhibited no such differences [71], suggesting HO-1 dysregulation is a COPD-specific hallmark rather than a universal smoking response. In this study, direct exosomal HO-1 quantification confirmed elevated HO-1 levels in COPD versus non-COPD murine lung Exos. Intravenous administration of Group B Exos into HO-1(−/−) mice elevated HO-1 expression in blood, lung, and brain tissues versus Group A Exos recipients, corroborating COPD-associated exosomal HO-1 enrichment.

Our experiments confirmed Exos traversal across the BBB, highlighting their central role in HO-1-mediated lung–brain signaling. In vivo imaging demonstrated BBB penetration of fluorescently labeled Exos within 24 h post-tail vein injection, with sustained cerebral accumulation through Day 5. Despite preferential pulmonary retention (higher lung vs. brain fluorescence, *p < *0.05 at Days 1/5; likely due to dense capillary networks or endothelial fenestrations), a subset of Exos breached the BBB into brain parenchyma. Group III exhibited faster cerebral fluorescence decline versus pulmonary, indicating rapid clearance of normal Exos in the brain. In contrast, Group IV showed comparable clearance rates between lung and brain, suggesting COPD-modified Exos prolong cerebral retention, facilitating HO-1 accumulation.

Although the total yield of Exos was lower in HO-1(+/+) exacerbation COPD mice than HO-1(+/+) mice, the cognitive impairment was induced only by HO-1(+/+) exacerbation COPD mice. Exos highlights a critical shift in their cargo composition rather than their abundance. This suggests that the pathophysiology of COPD drives a reprogramming of Exos content, leading to enhanced packaging of pathogenic factors such as HO-1.

Systemic HO-1 regulation was unequivocally demonstrated: HO-1 increases in blood Exos, lung Exos, and brain tissues occurred synchronously, implicating Exos in systemic HO-1 dissemination rather than organ-restricted delivery. This supports Exos not only as HO-1 transporters but also as amplifiers of pathological signaling via “Exos-target organ” positive feedback. Elevated blood exosomal HO-1 in Group IV versus Group I suggests its potential as an early biomarker for cerebral accumulation. Symmetrical HO-1 distribution in bilateral brain/lung tissues (Group III) excluded lateralization bias, confirming homogeneous Exos dispersion. Unlike prior studies focusing on exosomal miRNA/cytokine-mediated organ crosstalk [72], our work uniquely establishes HO-1 protein itself as a BBB-penetrating neuromodulator via Exos-dependent trafficking.

Additionally, we observed an intriguing phenomenon: Following exogenous Exos administration, HO-1 expression levels showed no significant disparity between left and right brain detection methods in Groups III/IV. In HO-1(−/−) mice receiving Group B Exos (Group IV), pulmonary HO-1 levels in homogenates were lower than those in lung-derived Exos, suggesting that exogenous HO-1 acquired by lung tissues is predominantly degraded locally, while the exosomal lipid bilayer may protect HO-1 from rapid metabolism, enabling sustained release. The BBB renders brain tissue more susceptible to exogenous HO-1. However, Group IV cerebral HO-1 levels remained comparable to PBS-injected C57 controls, indicating preferential HO-1 accumulation in the brain versus lungs. This validates our in vivo imaging findings: COPD-modified Exos exhibit prolonged cerebral retention, whereas pulmonary tissues efficiently metabolize HO-1. Restricted cerebral metabolic capacity and Exos persistence likely establish chronic neurotoxic pressure from HO-1 and iron metabolites.

Based on the integration of our experimental findings and existing theoretical frameworks, we propose the “HO-1 concentration-dependent neuroregulatory hypothesis” in COPD exacerbation mice: The impact of HO-1 on cognitive function exhibits a biphasic characteristic, with its biological effects determined by tissue concentration. Under physiological concentrations, HO-1 exerts antioxidant effects to protect neurons from ROS attack, potentially through mechanisms involving antioxidant promotion via metabolites [73, 74], reduction of tau expression and β-amyloid toxicity in neuroblastoma cells, and enhancement of neurotrophic factor production via the Nrf2/HO-1/BVR-A pathway [58–60]. In contrast, under pathological concentrations, persistently delivered HO-1 catalyzes excessive Fe^2+^ generation, which triggers lipid peroxidation storms via Fenton reactions, ultimately driving ferroptosis. Experimental evidence from this study supports this hypothesis: HO-1(−/−) mice lack endogenous HO-1, and the amount of HO-1 delivered by Exos in Group III is insufficient to compensate for this deficiency, resulting in overall HO-1 levels lower than those in wild-type mice (Group I). In cognitive tests, HO-1-deficient mice (Group II) showed no difference in cognitive performance compared to wild-type mice (Group I), likely due to full compensation by endogenous defense systems. Mice receiving low-load HO-1 Exos (Group III) exhibited cognitive function comparable to wild-type mice, whereas high-load HO-1 Exos-treated mice (Group IV) displayed cognitive decline, revealing a clear neuroprotective-toxic transition window for HO-1. This Exos-mediated HO-1 transport across the BBB may constitute a novel molecular basis for COPD-related cognitive impairment.

In summary, this study demonstrates that baseline HO-1 expression in wild-type mice forms a natural antioxidant defense network across blood Exos, lungs, and brain tissues. However, during COPD progression, pulmonary HO-1 expression significantly increases, leading to abnormal cerebral HO-1 accumulation via Exos-mediated trans-barrier transport—as confirmed by Exos tracking experiments showing fluorescently labeled Exos penetrating the BBB within 24 h of tail vein injection. Integrating published studies, we hypothesize that the “lung–brain HO-1 transport axis” drives COPD-related pathological cascades through: (1) Under normal conditions, HO-1 is lowly expressed and localized in the brain [30]. Studies in rat models show high levels of HO-1 mRNA detected in the hippocampus and cerebellum [75], indicating that cellular reserves of HO-1 are primed for rapid protein synthesis. Local oxidative stress (CS/hypoxia) directly induces upregulation of HO-1 expression in brain tissue; (2) continuous delivery of lung-derived Exos combined with COPD pathological modification prolongs the retention time of brain Exos, causing the total level of HO-1 in the brain to exceed the toxicity threshold; and (3) free Fe^2+^ produced by HO-1 catalysis induces ferroptosis. Sufficient evidence demonstrates the feasibility of targeting ferroptosis regulators for treating neurodegenerative diseases [76].

Based on these findings, future therapeutic strategies could focus on (1) developing an early warning system based on monitoring exosomal HO-1 load; (2) designing nanoscale iron chelators with BBB penetration capability; and (3) exploring spatiotemporally specific gene editing technologies to regulate HO-1 expression, ultimately achieving a paradigm shift from “blind concentration control” to “precise dose regulation.”

While this study has preliminarily revealed the concentration-dependent mechanism by which HO-1 regulates cognitive function through the Exos-mediated lung–brain axis, several aspects require further refinement: First, although the experimental hypothesis links the neurotoxic effects of HO-1 to ferroptosis, the absence of intervention experiments using ferroptosis-specific inhibitors prevents direct confirmation that HO-1-induced ferroptosis represents the core pathway underlying HO-1-mediated cognitive impairment. Further downstream experiments are needed to verify this relationship. Second, this study did not perform subsequent titration of HO-1 concentration thresholds. In future research, dynamic exploration of the concentration threshold for HO-1's dual effects could be achieved by correlating exosomal HO-1 load with cognitive scores. Third, we did not perform nanoparticle tracking analysis (NTA) for size distribution or comprehensive marker profiling beyond the data shown according to MISEV guidelines. Nevertheless, the robust functional outcomes observed in our in vivo experiments provide strong support for the biological activity of the Exos used in this study.

Another limitation of this study is the undefined cellular origin of the lung-derived Exos. While our data clearly demonstrate that Exos from WT-COPD lungs are sufficient to induce cognitive impairment, we cannot definitively specify whether they are predominantly secreted by alveolar epithelial cells, airway epithelial cells, macrophages, or other immune cells within the lung microenvironment. Pinpointing the exact source is technically challenging, as it requires sophisticated approaches such as cell-type-specific immunoprecipitation (e.g., using CD326 for epithelial cells or CD11b/CD45 for immune cells) in future studies. Determining the relative contributions of specific cell types to this pathogenic Exos pool represents an exciting and important direction for future research.

## 5. Conclusion

This study, based on a mouse model, confirms that COPD can induce cognitive impairment accompanied by abnormally elevated HO-1 expression in lung tissue. By integrating Exos tracing technology to construct a cross-organ transduction model, it reveals that lung-derived Exos may deliver HO-1 across organs and the BBB to brain tissue, leading to HO-1 accumulation in the brain and ultimately affecting cognitive function in mice. This discovery provides a critical theoretical basis for elucidating the interorgan interaction mechanisms underlying systemic complications of COPD. It also suggests that Exos-mediated HO-1 serves as a signaling hub in the lung–brain axis, emerging as a potential target for intervening in COPD-related neurodegenerative disorders. These findings lay the foundation for developing Exos-targeted neuroprotective strategies.

## Figures and Tables

**Table 1:** Quantitative analysis of HO-1 protein expression in lung tissues by Western blot.

Group	Group sample No.	HO-1 (gray value)^a^	β-Actin (gray value)^a^	HO-1/β-Actin (relative ratio)	Normalized HO-1^b^
Group A (WT-Con)	1	82,084	135,934	0.604	0.850
	2	96,638	123,408	0.783	1.103
	3	96,807	130,166	0.744	1.047
	Mean ± SD	91,843 ± 8465	129,836 ± 6512	0.710 ± 0.092	1.000 ± 0.131

Group B (WT-COPD)	1	119,128	138,613	0.859	1.210
	2	136,032	125,549	1.083	1.526
	3	133,588	129,799	1.029	1.449
	Mean ± SD	129,583 ± 9672	131,320 ± 6845	0.990 ± 0.124	1.328 ± 0.171

Statistical comparison^c^	—	—	—	—	*p* = 0.0047^∗∗^

^a^Gray values were quantified using ImageJ software by measuring the integrated density of protein bands. β-Actin served as the loading control to normalize protein loading.

^b^Normalized HO-1 expression was calculated by setting the mean value of Group A (WT-Con) as 1.0.

^c^Statistical analysis was performed using GraphPad Prism 10.0. Due to the small sample size (*n* = 3 per group), differences between Group A (WT-Con) and Group B (WT-COPD) were analyzed using the nonparametric Mann–Whitney *U* test (two-tailed). Significance was defined as ^∗∗^*p* < 0.01.

**Table 2:** Concentration of exosomes isolated from mouse lung tissues (as determined by EXOCET assay).

Group	OD value	Exos concentration (µg/µl)
WT-Con	0.9714	8.06
WT-COPD	0.8483	5.83

## Data Availability

The study does not contain any form of personal data, and our research data can be obtained by contacting the corresponding author.
